# Referral, Genetic Counselling, and *BRCA* Testing in the Manitoba High-Grade Serous Ovarian Cancer Population, 2004–2019

**DOI:** 10.3390/curroncol29120735

**Published:** 2022-11-30

**Authors:** Kelcey Winchar, Pascal Lambert, Kirk J. McManus, Bernie Chodirker, Sarah Kean, Kim Serfas, Kathleen Decker, Mark W. Nachtigal, Alon D. Altman

**Affiliations:** 1 Obstetrics, Gynecology and Reproductive Sciences, University of Manitoba, Winnipeg, MB R3A 1R9, Canada; 2 CancerCare Manitoba Research Institute, CancerCare Manitoba, Winnipeg, MB R3E OV9, Canada; 3 Epidemiology and Cancer Registry, CancerCare Manitoba, Winnipeg, MB R3E OV9, Canada; 4 Biochemistry and Medical Genetics, University of Manitoba, Winnipeg, MB R3E OV9, Canada; 5 Pediatrics and Child Health (Section of Genetics and Metabolism), University of Manitoba, Winnipeg, MB R3E OV9, Canada

**Keywords:** BRCA, genetic testing, genetic referral, high-grade serous ovarian cancer

## Abstract

**Simple Summary:**

This study was performed to better understand rates and factors that influence patients in accepting a referral to genetics or testing for genes that predispose them to ovarian cancer (*BRCA1/2*). Using multiple provincial databases and registries, the study team looked at data from 944 patients with high-grade ovarian cancer between 2004–2019. We found that the rate of genetic referrals fluctuated over time; however, the rate of genetic testing increased over the entire timeframe. Factors found to increase rates of referral and testing included age, cancer histology, history of oral contraceptive use, and family history of ovarian cancer. Increasing the rate of genetic testing will help patients and their health care team plan clinical management and treatment.

**Abstract:**

(1) Background: The primary objective of this study was to examine the rate of genetic referral, BRCA testing, and BRCA positivity amongst all patients with high-grade serous ovarian cancers (HGSOC) from 2004–2019. The secondary objective was to analyze secondary factors that may affect the rates of referral and testing. (2) Methods: This population-based cohort study included all women diagnosed with HGSOC using the Manitoba Cancer Registry, CervixCheck registry, *Medical Claims* database at Manitoba Health, the Hospital Discharge abstract, the Population Registry, and Winnipeg Regional Health Authority genetics data. Data were examined for three different time cohorts (2004–2013, 2014–2016; 2017–2019) correlating to practice pattern changes. (3) Results: A total of 944 patients were diagnosed with HGSOC. The rate of genetic referrals changed over the three timeframes (20.0% → 56.7% → 36.6%) and rate of genetic testing increased over the entire timeframe. Factors found to increase rates of referral and testing included age, histology, history of oral contraceptive use, and family history of ovarian cancer. Prior health care utilization indicators did not affect genetic referral or testing. (4) Conclusion: The rate of genetic referral (2004–2016) and *BRCA1/2* testing (2004–2019) for patients with a diagnosis of HGSOC increased over time. A minority of patients received a consultation for genetics counselling, and even fewer received testing for a *BRCA1/2*. Without a genetic result, it is difficult for clinicians to inform treatment decisions. Additional efforts are needed to increase genetics consultation and testing for Manitoban patients with HGSOC. Effects of routine tumour testing on rates of genetic referral will have to be examined in future studies.

## 1. Introduction

Genetic predisposition is a well-established risk and prognostic factor in patients with epithelial ovarian cancers (EOCs). The predominant histologic subtype of EOC is high-grade serous ovarian cancer (HGSOC), which includes serous ovarian, peritoneal, and fallopian tube cancers. Approximately 15% of HGSOC cases are attributed to a hereditary mutation, of which *BRCA1* and *BRCA2* are the most well-known genes [1,2,3,4,5,6,7]. Genetic testing has been recommended by all major oncologic societies for women with HGSOC. Despite these recommendations, the referral rate to genetic counselling has remained low [8,9]. To date, screening for EOC has proven ineffective [10,11,12]. Identification of patients and families at an increased risk of hereditary breast and ovarian cancer is an essential strategy for prevention and early treatment. Furthermore, with the advent of targeted synthetic lethal therapies, such a poly (ADP-ribose) polymerase (PARP) inhibitors, genetic testing to detect *BRCA1* and *BRCA2* mutations is becoming a crucial part of clinical practice for women diagnosed with EOC [13,14,15].

Several studies have examined the uptake of genetic testing for a germline *BRCA1* or *BRCA2* mutation for breast and ovarian cancer patients [16,17,18]. Referral rates to genetics for EOC patients ranged from 14–21%. Multiple factors contribute to these low referral rates, including reluctance to refer a patient to clinical genetics services or a patient’s reluctance to be referred. Within Manitoba, the Departments of Pathology and Gynecologic Oncology implemented strategies to improve genetic referral including a change in genetics referral criteria from age-based (i.e., ovarian cancer < 50 years old) to increased inclusion (i.e., all HGSOC) in 2007 and a recommendation for genetic counselling on the pathology reports for all HGSOC specimens beginning in 2014. Another major innovation came in 2016, when next-generation sequencing was implemented in Manitoba, drastically decreasing result turnover time from 12–18 months down to 4 months. During the study period, all cases of HGSOC and unclassified gynecologic and peritoneal epithelial cancers that required genetic testing were expected to be referred to genetic counselling services until 2018, when gynecologic oncologist-initiated BRCA testing was started.

The primary objective of this population-based study was to review all HGSOC patients from 2004–2019 to determine the rate of genetic referral, rate of testing for germline *BRCA1* and *BRCA2* mutations, and rate of BRCA positivity (i.e., an inactivating mutation in *BRCA1* or *BRCA2*). The secondary objective was to examine factors that may influence referral and testing to genetic counselling services, such as tumour characteristics, sociodemographic factors, treatment, personal history, and health care utilization.

## 2. Materials and Methods

### 2.1. Study Design

This was a population-based cohort study of women with a new diagnosis of HGSOC and unclassified EOC between 1 January 2004–31 December 2019 using linked health care databases (described in Data Sources) in Manitoba, Canada. This study was reviewed and approved by the Research Ethics Board at the University of Manitoba (approval number HS21788/H2018:190), the Health Information Privacy Committee (approval number HIPC 2018/2019-02), as well as the CancerCare Manitoba Research Resource Impact Committee (approval number RRIC2017-047).

### 2.2. Patients

The study cohort was comprised of women with a histologic diagnosis of serous ovarian or unclassified EOC between 2004–2019; the study period was divided into three cohorts (2004–2013; 2014–2016; 2017–2019) correlating with known practice changes, external to the genetics department, in 2014 and 2018. Patient inclusion was based on topography codes for EOC of the peritoneum, peritoneum not otherwise specified, overlapping lesion of the retroperitoneum and peritoneum, ovarian, and other unspecified female and genital organs. Patients were excluded if they were not a Manitoba resident at the time of diagnosis and did not have 30 months of Manitoba Health coverage prior to diagnosis.

### 2.3. Data Sources

Records from the following linked databases were employed to identify patient characteristics, sociodemographic factors, health care utilization, and outcome data:

1. Manitoba Cancer Registry (MCR), which maintains records on diagnosis and associated variables including age at diagnosis and death, histology, grade, topography, treatment-including surgery and chemotherapy, and postal code;
2. The MCR is a population-based registry that is legally mandated to collect and maintain accurate, comprehensive information about cancer diagnoses in Manitoba and has consistently shown to be of very high quality [19]. Postal code was used to identify income quintiles and the distance between the residence at diagnosis and the cancer treatment centre where the genetics referral program is located. Area-level average household income was determined by linking postal code at diagnosis from the CCR to Statistics Canada’s Postal Code Conversion File (PCCF) to identify the Canadian Census dissemination area (DA) in which an individual resided. Dates of ductal carcinoma in situ breast cancer diagnosis, invasive breast cancer, and cervical cancer diagnosis were used to determine when individuals were no longer eligible for screening;
3. CervixCheck maintains a population-based registry of all Pap tests and colposcopies performed in the province. CervixCheck Registry was used to determine Pap test dates for three years prior to the EOC diagnosis;
4. Medical Claims Database at Manitoba Health, which provides health care utilization information including uptake of screening mammograms, periodic health examinations, and physician encounters for two years prior to cancer diagnosis, to determine continuity of care;
5. Hospital Discharge Abstracts used to exclude visits in the Medical Claims database that were inpatient visits;
6. The Population Registry that provided data regarding coverage and cancellation data for the cohort;
7. The Winnipeg Regional Health Authority (WRHA)/Shared Health (SH) provided data including referral and testing as well as referral to physician specialty and BRCA test results.

The Charlson Comorbidity Index [20] was used to determine comorbidities identified in the Hospital Discharge Abstracts and Medical Claims Database during the 12 months prior to the EOC diagnosis.

All predictor variables measuring health care utilization were measured prior to diagnosis and excluded the 6-month period prior to diagnosis. Presence of a primary care physician consisted of two visits over a 2-year period (6 to 30 months prior to diagnosis) to the same physician or having a periodic health examination and were identified as one of the following: general practitioner, gynecologist, and internal medicine physician (for patients 60 years and older). Continuity of care, for those with three or more physician visits over a 2-year period (6 to 30 months prior to diagnosis), is defined as having at least 50% of physician visits from the same physician [21]. The uptake of Pap tests and mammograms was also used to evaluate health care utilization.

Our previous EOC study [22] provided additional information for patients referred to CancerCare Manitoba between 2004–2014, including gravida/parity, smoking status, history of oral contraceptive use, history of hormone replacement therapy, and family cancer history at the time of diagnosis.

### 2.4. Outcomes

Rates of referral, rates of testing, and rates of BRCA positivity over time were assessed using dates entered in the WRHA database. The follow-up period was until the end of 2020, allowing for an appointment one year after the last date of diagnosis. The mean follow-up time is calculated from the date of diagnosis to whatever came first, (1) referral or (2) death.

### 2.5. Statistical Analysis

Rates of referrals and testing at 1-year post diagnosis were calculated using cumulative incidence with death as a competing risk. Therefore, calculated rates indicate the first of two events: referral and death or testing and death. Time trends for time to referrals and testing for EOC cases diagnosed from 2004–2019 were analyzed using competing risk regression models, with death as a competing risk. Splines were used to account for the expected non-linear relationship between age and the outcomes. Competing risk regression models were used to determine predictors of referrals, appointments, and testing using the risk regression package in R. Data from the MCR and Manitoba Health were used as predictors for the 2004–2019 cohort. A sub-cohort was analyzed further by including EOC cases that were referred to CCMB from 2004 to 2014, with data from the MCR and from our previous study, “Investigating causes for poor outcome of ovarian cancer patients in Manitoba” (HIPC No 2014/2015-60; HREB H2012:145; RRIC #29-2012) [22]. Subgroup analyses including cervical and breast cancer screening history were limited to age ranges where screening was offered (e.g., Pap smears 21–70 years and mammogram 53–74 years), and health care utilization was only assessed for the initial 2004–2016 cohort.

## 3. Results

A total of 944 patients diagnosed with HGSOC or unclassified EOC were identified from the MCR between 2004–2019. Patient and tumour characteristics are presented in Table 1, with the study period divided into three cohorts (2004–2013; 2014–2016; 2017–2019) correlating with known practice changes. The mean age at diagnosis was 66.4 years, consistent across the cohorts. Reflecting the population distribution of Manitoba, most patients resided in Winnipeg (58.7%).

**Figure 1:**
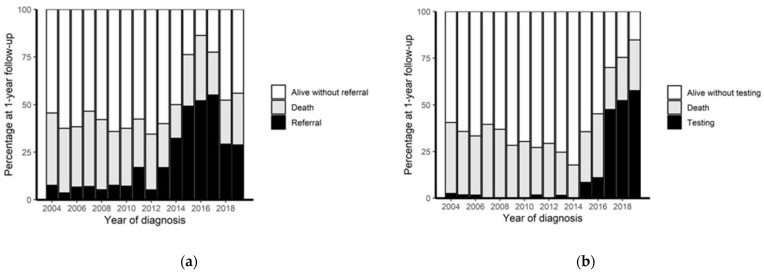
Percentage of genetic consultation at 1 year following diagnosis. Note: in 2014, pathology practice changed to include recommendation for genetics referral on submitted reports; in 2017, practice changed to allow gynecologic oncologist-initiated BRCA testing. (**a**) Percentage of patients with referrals to genetics within 1st year following diagnosis. (**b**) Percentage of patients with genetic results within 1st year following diagnosis.

Practice changes to gynecologic oncologist-initiated BRCA testing were implemented in 2018. Health care utilization did not differ from 2004 to 2016 (Table 2). Referrals to genetic counselling services occurred for 117/586 patients (20.0%) from 2004–2013, 110/194 patients (56.7%) from 2014–2016, and 60/164 patients (36.6%) from 2017–2019 (Figure 1a). Rates of genetic testing for germline *BRCA1/2* mutations also progressively increased in the 2014–2016 and 2017–2019 cohorts compared to the 2004–2013 cohort prior to practice changes (Figure 1b). The majority of those tested from referral (2004–2016; 86/114; 75.4%) were negative for any genetic mutation on the panel at the time (Table 3).

**Figure 2:**
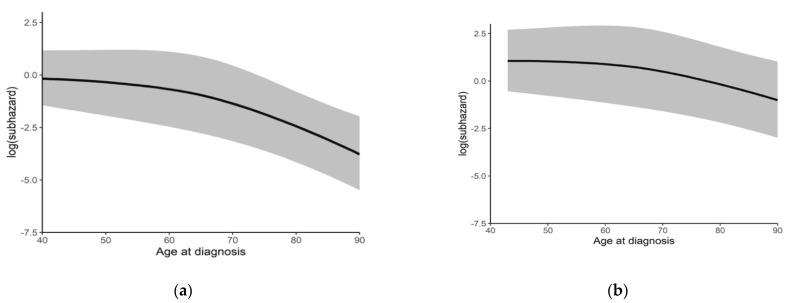
Association between age and genetics referral. (**a**) 2004–2013 (*n* = 117); (**b**) 2014–2016 (*n* = 110). Black lines indicate log (subhazards), with gray shading demarking the confidence interval.

Univariable and multivariable analyses were used to assess predictors of referral and genetic consultation in the cohort prior to gynecologic oncologist-initiated BRCA testing (2004–2016). The presence of a primary care physician, continuity of care, and prior breast or cervical cancer screening (predictors of health care utilization) did not influence referral rate for genetics consultation; 92.2% of patients in the 2004–2013 cohort and 88.1% in the 2014–2016 cohort had a primary care physician (Table 2). Factors shown to be associated with genetic consultation on multivariable analysis included age (*p* < 0.002), histology (*p* < 0.005), history of oral contraceptive use (*p* = 0.049), and a family history of EOC (*p* = 0.007) (Table A1). As shown in Figure 2, the effect of age is more pronounced in the 2004–2013 period (Figure 2a) compared to the 2014–2016 cohort (Figure 2b), with referral rates decreasing more rapidly with increasing age.

## 4. Discussion

Prior to change in practice in 2014 and again in 2018, referral to genetic testing for EOC patients was low. Over the study period, there was a gradual increase in referral rates until gynecologic oncologist-initiated tested was implemented, with the rates of testing for genetic mutations also increasing throughout. The strongest predictors of referral in the cohort included serous carcinoma histology, a younger age at diagnosis, and a positive family history for EOC.

Multiple studies have examined the reluctance of physicians to refer to genetic services; physician factors limiting referrals include lack of knowledge, lack of time, anticipated fear/distress of the patient, lack of appropriate family history, lack of awareness, and concern about discrimination of the patient [1,9,23,24,25,26,27,28]. This led to studies evaluating predictors of genetic referral. The predictive findings of age and change in practice seen in our study are reflected in the McGee et al. [8] and Febbraro et al. [17] retrospective studies of referral rates for genetic testing in the HGSOC population. Petzel et al. also analyzed predictors and uptake of referrals in Minnesota and found that 19% of patients receiving referrals were more likely to have a family history of EOC, be of a younger age at diagnosis, and have a serous histology. These findings were all attributed to an increasing awareness of the association between EOC and *BRCA1/2* mutations and tendencies to refer patients with traditional risk factors for EOC syndromes [29].

It was assumed that patients with a higher rate of health care utilization would be more inclined to accept referrals and further testing; in other words, patients that regularly take part in breast and cervical cancer screening or have a primary care physician were more likely to participate in other health promotion activities, such as genetic testing. However, health care utilization in the form of having a primary care physician, evidence of continuity of care, and prior breast cancer or cervical cancer screening all failed to predict genetic referrals or testing.

Of the 114 patients that underwent genetic testing as a part of their consultation, 21.1% tested positive for either *BRCA1* or *BRCA2* mutations. This is greater than the typical 15% of patients expected to have a hereditary *BRCA1/2* mutation associated with EOC [1,2,3,4,5,6,7]. The prevalence of mutations has been reported to be particularly high among women diagnosed in their forties (24.0%), in women with HGSOC histology (18%), and in women with a first-degree relative with breast or EOC (33.9%) [30].

Prompted by relatively low referral numbers, multiple studies examined strategies for improving genetic referral rates. McGee et al. (2017) retrospectively examined their referral rate for genetic testing in EOC patient populations and found a rate of 13%; by changing the referral approach to an “opt-out” strategy, referrals to genetics increased to 77% [8]. Petzel et al. (2014) analyzed a change in practice from standard referral to a systematically generated electronic referral practice, which increased rates from 17% to 30%; unfortunately, 40% of referred patients still did not see a genetic counsellor [1]. Kentwell et al. (2017) incorporated a genetic counsellor directly into the gynecologic oncology clinic and determined that their referral rates increased from 43% to 97% [23]. Schwartz et al. (2014) attempted telephone consultation to try to improve referral and testing rates [31]. Although the results were equivalent for satisfaction and costs were reduced, referral rates after telephone consultation were lower than in person (90.1% vs. 84.2%) [31]. Cohen et al. (2016) investigated the incorporation of clinical genetic representatives on gynecologic oncology tumour boards and found an increased referral rate from 26.7% to 51.7% [7]. Our study highlights how several practice changes (i.e., pathology reporting and gynecologic oncologist-initiated BRCA testing) can increase rates of referral and genetic testing. The importance of testing is crucial in a modern clinical context given the increasing use of targeted therapies (e.g., PARP inhibitors).

At the beginning of the study period, the protocol in Manitoba for referral to genetics screening consisted of receiving a pathology report and sending the referral to medical genetics, who would contact the patient to organize an appointment and provide testing. The reason for the increase in referrals to medical genetics seen during the 2014–2016 period was likely due to a change in practice of pathology reports; starting in 2014, the pathology report for patients with HGSOC included a footnote indicating that the patient should receive a referral to genetic counselling services.

In 2018, it also became possible for the gynecologic oncologists to directly send patients for *BRCA1/2* testing. This strategy did not require a genetics referral, and subsequently, the dates of these referrals were not collected in the genetics clinical database, and subsequently, genetic referrals decreased (Figure 1a). This explains the substantial undercount in the number of referrals collected. Due to this undercount, the 2017–2019 cohort could not be evaluated for predictors of referral. The rates of testing for genetic mutations continued to increase over 2017–2019 period, reflecting the change in practice.

Even more recently, reflex tumor testing for *BRCA1/2* mutations was done directly by the Department of Pathology, signalling patients be referred to medical genetics if they tested positive. Future studies will be required to determine whether these strategies result in increasing referral and screening.

Limitations of our study include that it was not possible to capture women who were referred to a genetic counselling appointment but did not attend the appointment or women who were referred at a later time in their treatment course. While women with previously poor health care utilization had no difference in genetics referral rates, it is possible that these patients may have been less likely to attend an appointment following referral. Unfortunately, this could not be assessed. We know from other published studies that there are multiple reasons why women may not want to attend a genetics referral, whether it be due to distance from appointments, lack of interest, fear, lack of family members that may benefit, and not perceiving any benefit to getting tested [9,32,33,34,35]. This may have had an impact on the amount of genetic testing performed despite patients receiving a referral. The study is also underpowered, as there are only about 110 events in each referral cohort. There are not enough referrals to include all predictors in the multivariable model, and thus, only the univariable significant variables were included in the multivariable models. Likewise, the number of patients receiving a referral and a testing result was too small to perform statistical analyses on the predictors of which patients received genetic testing. With the increasing numbers of referrals and patients receiving genetic testing towards the end of 2019, there may be additional benefit to analyzing patients beyond the current timeframe to improve the study power and allow those receiving testing to be evaluated. Additional practice changes, including *BRCA1* and *BRCA2* mutation testing by gynecologic oncologists and reflex tumor testing by the Department of Pathology, may also be evaluated in future years to determine their impact on detecting *BRCA1/2* positivity (i.e., rates). Northern patients were less likely to receive a referral than other Manitoba regional health authorities although the result was not significant, as the power may have been limited due to the small number of northern patients (3.5% of the total cohort).

## 5. Conclusions

In conclusion, rates of genetic referral (2004–2016) and *BRCA1/2* testing (2004–2019) for patients with a diagnosis of HGSOC increased over the course of the study. A family history of EOC, younger age at diagnosis, and serous histology were the strongest predictive factors for receiving a genetics referral. Still, a minority of patients received a consultation for genetics counselling, and even fewer received testing for a *BRCA1/2* genetic mutation. Without a result, it is difficult for clinicians to inform treatment decisions. This is also the first study examining health utilization behaviour and the association with genetic referral and testing, which did not show a significant association. Additional efforts are needed to increase genetics consultation and testing for Manitoban patients with HGSOC in order to provide patients with informed treatment options.

## Figures and Tables

**Table 1:** Manitoba HGSOC patient and tumour baseline characteristics, 2004–2019.

Diagnosis of HGSOC	Subgrouping	2004–2013 *n* = 586	2014–2016 *n* = 194	2017–2019 *n* = 164	Full Cohort *n* = 944
Age	Mean (SD)	66.2 (13.2)	66.7 (12.3)	66.6 (12.8)	66.4 (13.0)
Regional Health Authority	Interlake-Eastern	78 (13.3)	22 (11.3)	22 (13.4)	122 (12.9)
	Northern	23 (3.9)	4 (2.1)	8 (4.9)	35 (3.7)
	Southern Health	66 (11.3)	23 (11.9)	21 (12.8)	110 (11.7)
	Prairie Mountain	80 (13.7)	27 (13.9)	15 (9.1)	122 (12.9)
	Winnipeg	339 (57.8)	118 (60.8)	98 (59.8)	555 (58.8)
* Income	U4–U5	114 (19.5)	49 (25.3)	47 (28.7)	210 (22.2)
	U1–U3	241 (41.1)	68 (35.1)	54 (32.9)	363 (38.5)
	R4–R5	93 (15.9)	22 (11.3)	28 (17.1)	143 (15.1)
	R1–R3	133 (22.7)	50 (25.8)	33 (20.1)	216 (22.9)
	Missing	5 (0.9)	5 (2.6)	2 (1.2)	12 (1.3)
Charlson	0	325 (55.5)	109 (56.2)		434 (55.6)
	1	130 (22.2)	45 (23.2)		175 (22.4)
	2+	131 (22.4)	40 (20.6)		171 (21.9)
Grade	Moderately or poorly differentiated	166 (28.3)	8 (4.1)	2 (1.2)	176 (18.6)
	Undifferentiated	101 (17.2)	64 (33.0)	15 (9.1)	180 (19.1)
	Unknown	319 (54.4)	122 (62.9)	147 (89.6)	588 (62.3)
Histology	Serous carcinoma	323 (55.1)	128 (66.0)	114 (50)	565 (59.9)
	Unclassified epithelial	263 (44.9)	66 (34.0)	50 (30.5)	379 (40.1)
Stage	I	53 (9.0)	9 (4.6)	8 (4.9)	70 (7.4)
	II	62 (10.6)	12 (6.2)	9 (5.5)	83 (8.8)
	III	271 (46.2)	108 (55.7)	80 (48.8)	459 (48.6)
	IV	151 (25.8)	43 (22.2)	37 (22.6)	231 (24.4)
	Unknown	49 (8.4)	22 (11.3)	30 (18.3)	101 (10.7)

* U, urban; R, rural. Income quintiles from 1 (lowest) to 5 (highest).

**Table 2:** Health care utilization by HGSOC patients in Manitoba, 2004–2016.

Diagnosis of HGSOC		2004–2013 *n* = 586	2014–2016 *n* = 194
Presence of a primary care physician	Yes	540 (92.2)	171 (88.1)
	No	46 (7.8)	23 (11.9)
Continuity of care	Yes	421 (71.8)	132 (68.0)
	No	103 (17.6)	29 (14.9)
	Less than 3 visits	62 (10.6)	33 (17.0)
Breast screening *	Yes	168 (65.4)	54 (60.0)
	No	89 (34.6)	36 (40.0)
Cervical cancer screening **	Yes	185 (59.7)	58 (54.7)
	No	125 (40.3)	48 (45.3)

* Reported as breast screening 0.3–2.5 years prior to diagnosis in women aged 52.2 to 69 at date of ovarian cancer diagnosis. ** Reported as Pap test 0.3–3.5 years prior to diagnosis in women aged 24.5 to 69 at date of ovarian cancer diagnosis.

**Table 3:** Results of genetic testing (2004–2016), *n* = 114.

Result	*n* (%)
*BRCA1*	19 (16.7)
*BRCA2*	5 (4.4)
*BRCA* not otherwise specified	2 (1.8)
Positive	1 (0.9)
*PALB2*	1 (0.9)
No mutation	86 (75.4)

## Data Availability

Data was derived from the Manitoba Cancer Registry, CervixCheck registry, *Medical Claims* database at Manitoba Health, the Hospital Discharge abstract, the Population Registry, and Winnipeg Regional Health Authority genetics data. None of these databases are publicly available.

## Appendix A

**Table A1:** Predictors of genetics referral.

		Diagnosis Years 2004–2013, *n* = 117 Referrals						Diagnosis Years 2014–2016, *n* = 110 Referrals					
		Univariable Analysis			Multivariable Analysis			Univariable Analysis			Multivariable Analysis		
		SHR	95% CI	*p*	SHR	95% CI	*p*	SHR	95% CI	*p*	SHR	95% CI	*p*
Region	Northern	Ref	Ref	0.363				Ref	Ref	0.820			
	Interlake-Eastern	3.11	0.73–13.31					3.27	0.47–22.94				
	Southern Health	1.61	0.35–7.46					2.99	0.41–21.63				
	Prairie Mountain	2.46	0.57–10.46					2.98	0.43–20.86				
	Winnipeg	2.57	0.63–10.41					3.20	0.48–21.25				
Income	U4–U5	1.39	0.88–2.19	0.154				1.72	1.08–2.74	0.022			
	U1–U3	Ref	Ref					Ref	Ref				
	R4–R5	2.57	1.35–4.91	0.004				0.68	0.32–1.42	0.301			
	R1–R3	Ref	Ref					Ref	Ref				
Grade	Unknown	0.92	0.57–1.48	0.010				0.96	0.36–2.57	<0.001	2.27	0.65–7.90	0.278
	Undifferentiated	3.02	1.88–4.83					2.31	0.86–6.20		2.52	0.78–8.16	
	Moderately or poorly differentiated	Ref	Ref					Ref	Ref		Ref	Ref	
Histology	Serous carcinoma	4.49	2.77–7.27	<0.001				4.40	2.60–7.43	<0.001	2.33	1.30–4.18	0.005
	Unclassified epithelial	Ref						Ref	Ref		Ref	Ref	
Stage	Unknown	1.43	0.57–3.59	0.010				0.44	0.17–1.17	0.001	0.49	0.01–1.30	0.168
	I–II	2.39	1.29–4.43					1.91	0.94–3.89		1.15	0.53–2.45	
	III	2.44	1.40–4.24					2.04	1.16–3.59		1.27	0.71–2.29	
	IV	Ref						Ref	Ref		Ref	Ref	
Presence of a primary care physician	Yes	1.34	0.61–2.94	0.458				0.75	0.44–1.3	0.312			
	No	Ref						Ref	Ref				
Continuity of care	Less than 3 visits	1.44	0.69–3.03	0.457				1.44	0.75–2.76	0.199			
	Yes	1.39	0.82–2.36					0.94	0.54–1.64				
	No	Ref						Ref	Ref				
Charlson index	0	1.29	0.80–2.10	<0.555				1.71	1.03–2.85	0.069			
	1	1.14	0.64–2.02					1.19	0.63–2.24				
	2+	Ref						Ref	Ref				
Gravida	Missing	0.33	0.15–0.75	<0.001									
	4+	0.86	0.45–1.65										
	1–3	1.61	0.93–2.79										
	0	Ref											
Parity	Missing	0.36	0.15–0.75	<0.001									
	4+	0.59	0.45–1.65										
	1–3	1.40	0.86–2.30										
	0	Ref											
History of oral contraceptive use	Missing	0.56	0.34–0.92	<0.001	0.68	0.42–1.11	0.049						
	Yes	1.91	1.18–3.10		1.15	0.70–1.88							
	No	Ref	Ref		Ref	Ref							
History of HRT use	Missing	0.57	0.38–0.85	0.021									
	Yes	0.88	0.54–1.42										
	No	Ref	Ref										
Smoking	Missing	1.45	0.52–4.03	0.227									
	Current	2.10	0.69–6.44										
	Former	2.05	0.70–5.94										
	Never	Ref	Ref										
Alcohol use	Missing	0.34	0.18–0.62	<0.001									
	Current	1.35	0.90–2.01										
	Former	1.42	0.45–4.43										
	Never	Ref	Ref										
Family history of ovarian cancer	Yes	3.40	2.18–5.30	<0.001	1.96	1.20–3.20	0.007						
	No	Ref	Ref		Ref	Ref							
Breast cancer screening *	Yes No	1.35 Ref	0.80–2.25 Ref	0.260				1.33 Ref	0.80–2.21 Ref	0.272			
Cervical cancer screening **	Yes No	1.57 Ref	1.02–2.42	0.039				1.45 Ref	0.92–2.28 Ref	0.107			

* Reported as breast screening 0.5 to 2.5 years prior to diagnosis in women aged 52.2 to 69 at date of ovarian cancer diagnosis. ** Reported as pap test 0.5 to 3.35 years prior to diagnosis in women aged 24.5 to 69 at date of ovarian cancer diagnosis.
